# Types and Diagnosis of Childhood Intellectual Disabilities: Advancing Accuracy for Better Outcomes

**DOI:** 10.3390/children12121585

**Published:** 2025-11-22

**Authors:** Rasha Babiker, Manal M. Sami, Haifa K. Ahmed, Rasha A. Salama

**Affiliations:** 1 Department of Physiology, RAK College of Medical Sciences, RAK Medical and Health Sciences University, Ras Al Khaimah 11172, United Arab Emirates; 2 Department of Pathology, RAK College of Medical Sciences, RAK Medical and Health Sciences University, Ras Al Khaimah 11172, United Arab Emirates; manal@rakmhsu.ac.ae; 3 Department of General Education, Psychology, RAK College of Medical Sciences, RAK Medical and Health Sciences University, Ras Al Khaimah 11172, United Arab Emirates; haifa@rakmhsu.ac.ae; 4 Department of Community Medicine, RAK College of Medical Sciences, RAK Medical and Health Sciences University, Ras Al Khaimah 11172, United Arab Emirates; rasha.aziz@rakmhsu.ac.ae

**Keywords:** intellectual disability, intellectual developmental disorder, disorders of intellectual development, adaptive functioning, developmental surveillance, cognitive assessment, Vineland-3, ABAS-3, chromosomal microarray, exome sequencing, early intervention, DSM-5-TR, ICD-11

## Abstract

**Highlights:**

What are the main findings?

• Updated DSM-5-TR/ICD-11 criteria highlight adaptive functioning and standardized assessments for accurate ID diagnosis.
• Genetic testing greatly improves identifying the underlying causes of ID.

What is the implication of the main finding?

• Earlier and precise diagnosis enables timely interventions and better individualized outcomes.
• Aetiology-based diagnosis strengthens prognosis, family counseling, and targeted management.

**Abstract:**

Intellectual disability (ID)—termed intellectual developmental disorder in DSM-5-TR and disorders of intellectual development in ICD-11—is a heterogeneous spectrum of neurodevelopmental conditions characterized by impairments in general intellectual abilities and adaptive functioning, with onset during the developmental period. Precise and early recognition of ID can alter developmental trajectories by clarifying prognosis, guiding targeted genetic and medical investigations, initiating time-sensitive interventions, preventing diagnostic overshadowing, and informing educational planning and family counseling. This narrative review synthesizes contemporary diagnostic standards from DSM-5-TR and ICD-11, recent updates in epidemiology, and an aetiology-oriented framework for classifying “types” of intellectual disability. It further outlines current best practices in diagnostic work-up—including developmental surveillance and screening, standardized cognitive and adaptive assessments, genomic testing (microarray, exome/genome sequencing, and syndrome-specific assays), selective metabolic studies, and neuroimaging or EEG when indicated. Additionally, the review explores culturally responsive and equitable assessment, special diagnostic contexts (such as profound impairment, sensory and motor confounds, and bilingualism), and the frequent co-occurrence of other neurodevelopmental and medical disorders. The discussion concludes with practical, stepwise diagnostic pathways that clinicians can readily apply and anticipates emerging frontiers—such as genomic medicine and digital phenotyping—that promise to enhance diagnostic yield and clinical utility in the near future.

## 1. Introduction

According to the Diagnostic and Statistical Manual of Mental Disorders, Fifth Edition, Text Revision (DSM-5-TR), the condition termed Intellectual Developmental Disorder (Intellectual Disability) is diagnosed when three criteria are met: (i) deficits in intellectual functions identified through individually administered tests; (ii) concurrent limitations in adaptive functioning that restrict independence across conceptual, social, and practical domains; and (iii) onset during the developmental period [1]. In both DSM-5-TR and the International Classification of Diseases, 11th Revision (ICD-11), severity is categorized as mild, moderate, severe, or profound [2].

For clarity, the terms Intellectual Disability (ID), Intellectual Developmental Disorder (IDD) (DSM-5-TR), and Disorders of Intellectual Development (ICD-11) are used interchangeably in this review. The abbreviation ID is retained throughout for consistency with current clinical and research usage.

**Figure 1:**
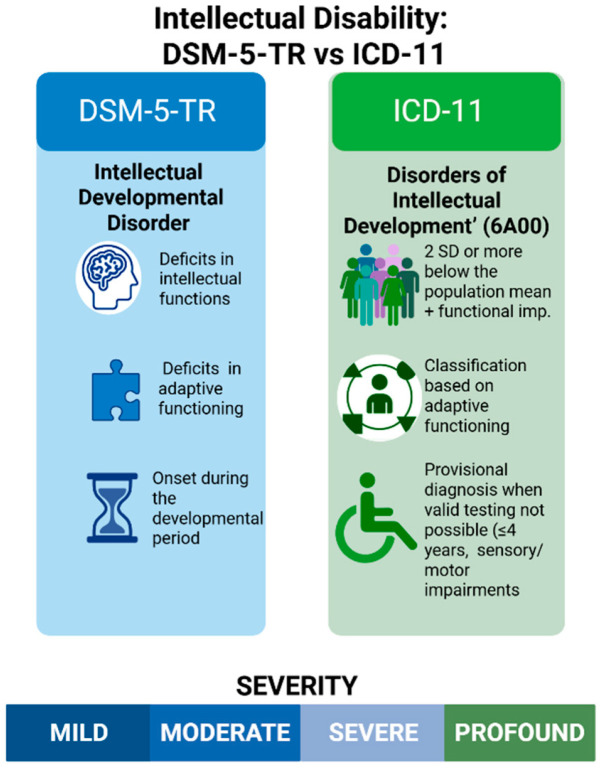
This is a figure that compares the diagnostic criteria for Intellectual Disability according to the DSM-5-TR (Diagnostic and Statistical Manual of Mental Disorders, Text Revision) and the ICD-11 (International Classification of Diseases, 11th Revision). Created in BioRender. Babiker, R. (2025) https://BioRender.com/ta2y687.

Crucially, this classification emphasizes adaptive functioning rather than IQ alone, as everyday competence provides a more accurate reflection of autonomy and social participation [3]. ICD-11 refers to the condition as Disorders of Intellectual Development (6A00) and requires standardized assessments demonstrating performance approximately two standard deviations below the population mean, accompanied by functional impairment [4]. To accommodate developmental variability, ICD-11 introduces a provisional category for very young children (typically under four years) or for those whose severe sensory or motor impairments preclude valid testing [5]. The 2024 Clinical Descriptions and Diagnostic Requirements (CDDR) further refine these criteria, offering harmonized thresholds suitable for cross-cultural application [6]. Figure 1 visually compares the diagnostic criteria for intellectual disability across DSM-5-TR and ICD-11, highlighting their convergences and nuanced distinctions.

Global point prevalence estimates for intellectual disability in the general population range from 1% to 3%, with variability largely reflecting methodological and definitional differences across studies [7]. Analyses based on GBD 2019 data suggest that approximately 1.4% of the global population was affected in 2019, though considerable regional variation persists. Complementary UNICEF and GBD syntheses highlight the broader spectrum of childhood developmental disabilities—encompassing hundreds of millions of children worldwide—within which intellectual disability constitutes a significant subset [8]. In U.S. surveillance data (NHIS 2019–2021), diagnosed intellectual disability among children aged 3–17 years remained stable at approximately 1.7–2.2%, despite an overall rise in developmental disability diagnoses [9]. Such variation reflects disparities in ascertainment, diagnostic criteria, and health-system infrastructure rather than biological differences. Detection is strongly influenced by developmental monitoring and screening policies. The American Academy of Pediatrics (AAP) currently recommends standardized developmental screening at 9, 18, and 30 months and autism-specific screening at 18 and 24 months, embedded within continuous developmental surveillance—an approach that also facilitates the earlier recognition of many children who are later diagnosed with intellectual disability [10]. Therefore, this review aims to comprehensively synthesize contemporary understanding of the classification and diagnostic frameworks for childhood intellectual disabilities, emphasizing how refinements in nosology and assessment can strengthen early recognition, inform intervention planning, and ultimately improve long-term developmental outcomes.

### 1.1. Types of Intellectual Disability: A Clinically Useful, Aetiology-Oriented Framing

**Figure 2:**
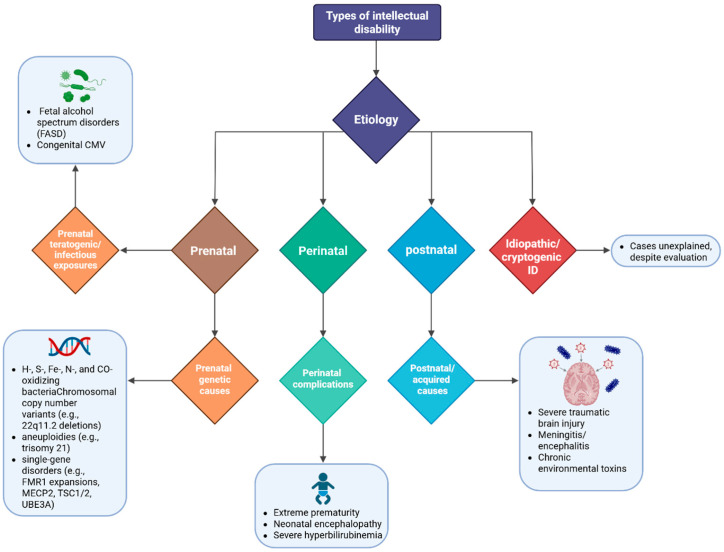
This figure is an overview of intellectual disability etiology showing major categories: prenatal, perinatal, postnatal, and idiopathic causes. Created in BioRender. Babiker, R. (2025) https://BioRender.com/31lnob7.

Because DSM-5-TR and ICD-11 classify intellectual disability only by severity, clinicians and researchers often adopt an aetiology-based framework to enhance diagnostic clarity and management. This structure organizes causes by timing—prenatal, perinatal, and postnatal—and by causal category, including genetic, environmental, and acquired factors. Such an approach not only facilitates comprehensive diagnostic work-ups but also informs recurrence risk counselling and guides individualized management strategies [11]. (Figure 2 provides a visual synthesis of these major aetiologic categories—prenatal, perinatal, postnatal, and idiopathic—illustrating how temporal classification supports diagnostic reasoning and prevention planning.)

#### 1.1.1. Prenatal Genetic Causes

Chromosomal copy number variants (e.g., 22q11.2 deletions), aneuploidies (e.g., trisomy 21), and single-gene disorders (such as FMR1 expansions, MECP2, TSC1/2, and UBE3A mutations) account for a significant proportion of moderate-to-severe intellectual disability. Contemporary genomic practice recognizes that exome and genome sequencing substantially increase diagnostic yield beyond that achieved with chromosomal microarray, often reshaping surveillance protocols and refining family counselling [12].

#### 1.1.2. Prenatal Teratogenic and Infectious Exposures

Fetal alcohol spectrum disorders (FASDs) remain under-recognized despite their prevalence and preventability. Updated American Academy of Pediatrics (AAP) guidance emphasizes multidisciplinary diagnostic assessment and meticulous documentation of prenatal exposure. Congenital cytomegalovirus (CMV) infection—the most common congenital infection globally, occurring in approximately one in every 200 births in high-income regions—represents a major cause of sensorineural hearing loss and neurodevelopmental disability. The long-term cognitive and functional sequelae of CMV vary considerably depending on whether the infant was symptomatic at birth [13,14].

Beyond CMV, other congenital infections within the TORCHES spectrum—namely *Toxoplasma gondii*, *Rubella virus*, *Treponema pallidum*, *Herpes simplex virus*, and emerging pathogens such as *Zika virus*—are also established causes of neurodevelopmental impairment and intellectual disability. Their prevention through maternal screening, vaccination (for rubella), and infection control during pregnancy remains a cornerstone of global public health efforts to reduce congenital neurodisability [15].

#### 1.1.3. Perinatal Complications and Postnatal Causes

Perinatal factors such as extreme prematurity, neonatal encephalopathy, and severe hyperbilirubinemia significantly elevate the risk of intellectual impairment. These are best conceptualized through the lens of early brain injury and disrupted neuronal connectivity, though advances in neonatal intensive care continue to mitigate risk [16].

Postnatal or acquired causes include severe traumatic brain injury, meningitis or encephalitis, and chronic exposure to environmental toxins, particularly lead. Recognizing the neurotoxic impact of even low-level exposure, the U.S. Centers for Disease Control and Prevention (CDC) lowered the blood lead reference value to 3.5 µg/dL in 2021 to prompt earlier intervention [17].

#### 1.1.4. Idiopathic or Cryptogenic Intellectual Disability

Despite extensive diagnostic evaluation, a subset of cases remains unresolved and is thus classified as idiopathic or cryptogenic. However, this fraction continues to decline as genomic technologies advance and periodic reanalysis of previously nondiagnostic exome data yields new gene–disease associations and additional molecular diagnoses over time [18].

## 2. Diagnosis

Establishing an accurate diagnosis of intellectual disability is pivotal, as it directly determines management strategies, intervention pathways, and long-term outcomes [19]. Identifying a specific etiology, whether genetic, teratogenic, or acquired, guides clinical care in several critical ways: it clarifies the natural history of the condition, identifies comorbidity risks such as epilepsy or cardiac and renal anomalies within syndrome-specific contexts, and informs anticipatory medical surveillance [20]. A precise diagnostic conclusion also facilitates targeted therapeutic interventions, such as the use of mTOR inhibitors in tuberous sclerosis, streamlines educational and behavioral planning, and provides families with accurate recurrence-risk counselling [21]. Moreover, prompt and confident diagnostic confirmation helps prevent prolonged diagnostic delays and repeated evaluations across multiple providers, reducing the emotional and financial strain often experienced by families seeking answers [22].

**Figure 3:**
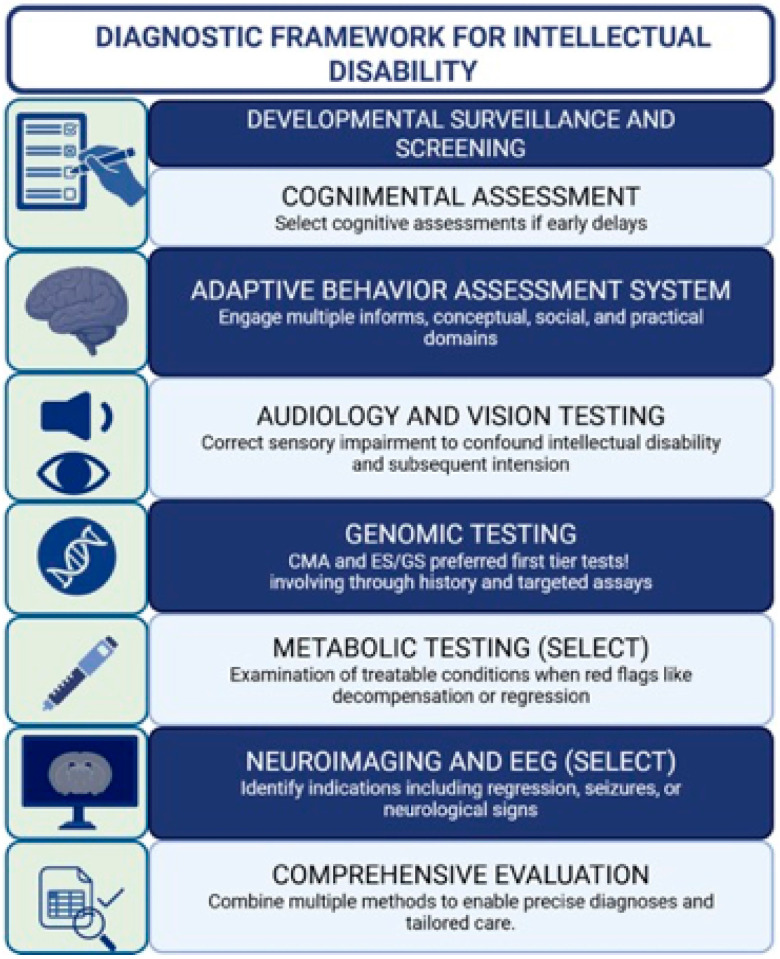
This figure summarizes the diagnostic framework for intellectual disability from screening to comprehensive evaluation. It involves developmental, cognitive, and adaptive assessments, followed by sensory, genetic, metabolic, and neuroimaging tests as indicated. Integration of findings enables accurate diagnosis and personalized management. Created in BioRender. Babiker, R. (2025) https://BioRender.com/yq08oxp.

Diagnosis begins even before formal testing, with the developmental assessment itself (Figure 3). Attentive listening to parental concerns, detailed review of developmental milestones, appraisal of prenatal and perinatal exposures, and awareness of social determinants often yield the earliest indicators of developmental delay [23,24]. The American Academy of Pediatrics (AAP) recommends standardized developmental screening at 9, 18, and 30 months, along with autism-specific screening at 18 and 24 months, integrated into continuous developmental surveillance at every well-child visit. Importantly, referral for early intervention should proceed without delay, even in the absence of a confirmed aetiologic label [25]. (Figure 3 summarizes this diagnostic framework—from developmental screening to comprehensive evaluation—illustrating how each assessment domain contributes to timely recognition and personalized management.)

When standardized testing is undertaken, cognitive assessment forms the cornerstone of diagnostic evaluation. For infants and toddlers, the Bayley Scales of Infant and Toddler Development, Fourth Edition (Bayley-4) remains the gold standard, assessing cognitive, language, and motor domains from 16 days to 42 months of age [26]. In preschool years, the Wechsler Preschool and Primary Scale of Intelligence, Fourth Edition (WPPSI-IV), offers early cognitive profiles, while the Wechsler Intelligence Scale for Children, Fifth Edition (WISC-V), is standard for ages 6–16, providing index scores aligned with DSM-5-TR and ICD-11 domains [27]. For children with significant language or hearing limitations, or those from linguistically diverse backgrounds, the Leiter International Performance Scale, Third Edition (Leiter-3), serves as a fully nonverbal alternative, spanning ages 3–75 [28]. Selection of the most appropriate instrument should be individualized, taking into account linguistic, sensory, and motor factors, as well as cultural context. Interpretation of results must extend beyond numerical scores to include qualitative observations and performance validity [29].

Because the classification of severity in intellectual disability is primarily based on adaptive functioning rather than IQ scores alone, every comprehensive evaluation should incorporate a norm-referenced measure of adaptive behaviour. The Vineland Adaptive Behavior Scales, Third Edition (Vineland-3), and the Adaptive Behavior Assessment System, Third Edition (ABAS-3), are among the most widely used instruments, each providing standardized domain and composite scores across conceptual, social, and practical areas of functioning. To ensure accuracy, information should be gathered from multiple informants—such as parents, teachers, and caregivers—as reliance on a single rater can introduce bias and may not fully capture the child’s performance across different settings [30].

Audiologic and visual assessments are foundational to any diagnostic process. Unrecognized sensory impairment can mimic or exacerbate cognitive deficits, and since interventions in this domain are often actionable, hearing and vision testing should precede or accompany cognitive evaluation [31].

The genomic era has revolutionized diagnostic pathways. The American College of Medical Genetics and Genomics (ACMG) now recommends exome or genome sequencing (ES/GS) as a first- or second-tier test for children presenting with developmental delay, congenital anomalies, or intellectual disability [32]. These techniques demonstrate higher diagnostic yield and greater cost-effectiveness when implemented early, outperforming chromosomal microarray (CMA), though CMA continues to hold value as an initial investigation. Fragile X testing remains crucial, particularly in males or where family history is suggestive [33]. The 2025 AAP clinical report offers a structured algorithm integrating comprehensive history-taking, physical examination, CMA, ES/GS, and phenotype-directed assays such as methylation testing for Prader–Willi or Angelman syndromes, and MECP2 analysis in girls with Rett-like regression [34,35]. Periodic reanalysis of previously nondiagnostic exomes is strongly encouraged as gene–disease associations continue to expand. Evidence from real-world studies confirms that exome sequencing not only enhances diagnostic yield but also produces tangible improvements in medical management, especially in conditions requiring syndrome-specific surveillance [36].

Metabolic testing, though no longer routinely indicated for all cases, remains essential when specific red flags are present—such as episodic metabolic decompensation, developmental regression, multisystem involvement, or consanguinity. Many contemporary protocols now employ a “screen-for-treatable” strategy to ensure that rare but actionable disorders are not missed [37].

Neuroimaging and electroencephalography (EEG) play targeted but important roles. Brain MRI is recommended when neurological abnormalities, seizures, atypical head growth, or developmental regression are observed, while EEG should be performed in the presence of suspected epilepsy or unexplained paroxysmal episodes. Diagnostic yield is maximized when these investigations are guided by neurological examination and phenotypic cues, rather than applied indiscriminately [38].

Environmental and teratogenic factors must also be considered as part of a comprehensive evaluation. The CDC currently defines a blood lead reference value of 3.5 µg/dL as the threshold for clinical intervention. Fetal alcohol spectrum disorders (FASDs) require multidisciplinary assessment following updated AAP diagnostic frameworks. Congenital cytomegalovirus (CMV) should be a strong consideration in infants presenting with hearing loss, microcephaly, or characteristic neuroimaging findings. Several regions have initiated universal or targeted newborn CMV screening programs to facilitate early antiviral therapy and structured audiological follow-up [17,39].

Collectively, this integrative diagnostic framework underscores that the goal of diagnosis extends far beyond classification—it is the key to transforming care through earlier recognition, precision in assessment, and equitable access to timely interventions.

Figure 3 illustrates the sequential diagnostic pathway, emphasizing the integration of developmental surveillance, cognitive and adaptive testing, and tiered investigations to achieve timely and precise diagnosis. This helps clinicians visualize how each assessment domain contributes to a comprehensive diagnostic picture.

By combining structured developmental surveillance, robust cognitive and adaptive assessments, early genetic testing, and judicious use of metabolic, neuroimaging, and environmental evaluations, clinicians can achieve earlier and more precise diagnoses. This approach directly improves outcomes by enabling tailored medical care, timely interventions, and equitable access to services for children and families affected by intellectual disabilities [40,41].

### 2.1. Differential Diagnosis and Frequent Co-Occurrences

Intellectual disability (ID) seldom occurs in isolation; it is frequently associated with other neurodevelopmental and medical conditions. Commonly co-occurring disorders include autism spectrum disorder (ASD), attention-deficit/hyperactivity disorder (ADHD), language impairments, epilepsy, and motor coordination disorders. Recognizing and delineating these overlapping presentations is essential to prevent diagnostic overshadowing, a phenomenon in which the presence of one condition obscures recognition of another [42].

Recent U.S. surveillance data indicate that approximately 40% of eight-year-old children diagnosed with ASD also meet IQ-based criteria for ID, underscoring the reciprocal need to evaluate both cognitive and adaptive functioning in autism assessments and to screen for autism traits among children already diagnosed with ID [43]. In addition, unrecognized hearing or vision impairments, as well as severe speech–motor limitations, may artificially lower cognitive test scores, thus mimicking intellectual impairment. Comprehensive sensory evaluations and the use of suitable accommodations—such as nonverbal cognitive measures (e.g., Leiter-3) and augmentative or alternative communication supports—are therefore critical to ensure valid assessment outcomes [44,45].

Effective differential diagnosis requires a cohesive, multidisciplinary approach that integrates neurological, psychological, and developmental expertise. Such collaboration allows clinicians to capture the interplay between neurodevelopmental disorders, medical comorbidities, and sensory or motor constraints, ultimately yielding a precise functional profile that guides both intervention and prognostic planning [46].

### 2.2. Special Diagnostic Scenarios

Certain clinical situations necessitate adapted diagnostic approaches to ensure validity and fairness. Profound intellectual impairment or very early developmental age presents particular challenges, as conventional IQ testing may not provide reliable results. In these cases, developmentally anchored instruments such as the Bayley Scales of Infant and Toddler Development, Fourth Edition (Bayley-4), are preferred. The ICD-11 also allows the use of a provisional category in situations where formal testing cannot be appropriately conducted due to age or severe sensory and motor impairments [47,48]. Diagnostic emphasis in such instances should shift toward evaluating adaptive behaviour, identifying coexisting medical conditions, and ensuring immediate access to early intervention services while the etiologic investigation—most often genetic—proceeds in parallel [49].

In bilingual and culturally diverse children, diagnostic accuracy relies heavily on fairness and contextual sensitivity. Instruments that minimize linguistic demands, such as the Leiter-3, are recommended, along with the engagement of trained interpreters and the collection of collateral data from multiple environments, including home, school, and community. This methodology aligns with DSM-5-TR’s emphasis on equitable assessment practices and the World Health Organization’s Clinical Descriptions and Diagnostic Requirements (CDDR), both of which prioritize cross-cultural validity to reduce the risk of misclassification [50,51].

When sensory or motor confounds are present, collaboration with audiologists and ophthalmologists is essential. Testing environments should be adapted accordingly, and when fine-motor output is substantially limited, clinicians should select assessment tools that minimize motor demands. Observational methods and dynamic, learning-potential assessments—used alongside standardized testing—can provide a more authentic representation of a child’s cognitive and adaptive capacities [52].

**Figure 4:**
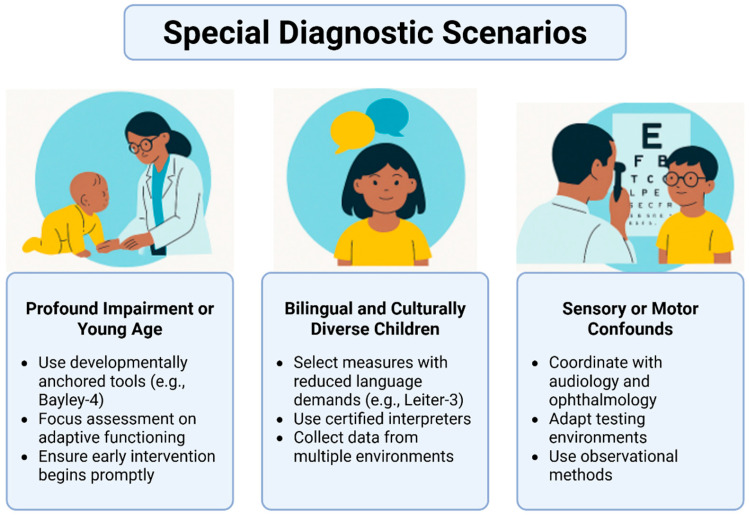
The special diagnostic scenarios for assessing profound impairment, bilingual and culturally diverse children, and sensory or motor confounders. Created in BioRender. Babiker, R. (2025) https://BioRender.com/39fe15u.

Figure 4 summarizes adapted diagnostic strategies for complex presentations such as profound impairment, bilingual or culturally diverse children, and cases with sensory or motor confounds, underscoring the need for fairness and individualized assessment.

### 2.3. From Diagnosis to Outcome

Accurate and timely diagnosis forms the cornerstone of effective care, as it profoundly influences both immediate interventions and long-term developmental outcomes. Early identification facilitates prompt access to early intervention services, individualized educational programs, and structured family support systems, all of which are known to markedly improve developmental trajectories and adaptive functioning. Moreover, a precise diagnosis enables syndrome-specific surveillance, allowing clinicians to anticipate and monitor comorbidities such as epilepsy, cardiac anomalies, and metabolic disturbances before they become clinically apparent [53,54].

Timely diagnosis also mitigates the emotional and financial toll of prolonged diagnostic odysseys by reducing unnecessary or repetitive investigations. Importantly, an established etiologic diagnosis allows affected children to be enrolled in condition-specific registries and clinical trials, thereby advancing research and opening opportunities for participation in emerging targeted therapies. For families, genetic clarification provides essential recurrence-risk counseling, supporting informed reproductive decision-making and facilitating long-term psychosocial adjustment [55,56].

Consensus statements increasingly affirm that genetic diagnoses directly influence care by refining surveillance strategies, guiding therapeutic planning, and informing family counseling. Furthermore, evidence suggests that genetic testing is most cost-effective when integrated early in the diagnostic process, rather than after exhaustive conventional investigations. Ultimately, diagnostic timeliness transforms the process from a purely descriptive exercise into a dynamic, action-oriented tool that enhances intervention outcomes, ensures equitable access to care, and improves the overall quality of life for children and their families [57].

### 2.4. Practical, Stepwise Pathway You Can Implement Now

A structured, stepwise diagnostic pathway ensures precision, efficiency, and continuity of care in evaluating intellectual disability. The process begins with a comprehensive medical history and physical examination, encompassing detailed prenatal, perinatal, and postnatal exposures, as well as a thorough family history to identify potential hereditary or environmental factors. This foundation is complemented by developmental surveillance and standardized screening, together with audiologic and visual evaluations, since uncorrected sensory impairments may mimic or aggravate cognitive limitations [58,59].

Cognitive and adaptive assessments should then be tailored to the child’s developmental level. The Bayley Scales of Infant and Toddler Development, Fourth Edition (Bayley-4), remain optimal for infants and toddlers, while the Wechsler Preschool and Primary Scale of Intelligence, Fourth Edition (WPPSI-IV), and the Wechsler Intelligence Scale for Children, Fifth Edition (WISC-V), are appropriate for preschool and school-age groups, respectively. Nonverbal instruments such as the Leiter International Performance Scale, Third Edition (Leiter-3), are particularly valuable for children with limited speech or hearing. Assessment of adaptive functioning using the Vineland-3 or ABAS-3 is indispensable, as contemporary classifications determine severity primarily through adaptive abilities rather than IQ alone [60,61].

Genomic testing should be initiated in accordance with best-practice guidelines. Standard evaluation includes chromosomal microarray (CMA) and exome or genome sequencing (ES/GS), supplemented by Fragile X testing when indicated, particularly in males or where family history suggests risk. Additional targeted studies—such as methylation assays for Prader–Willi or Angelman syndromes—are warranted when the phenotype is suggestive. Given rapid advances in genomic interpretation, periodic reanalysis of previously nondiagnostic exomes is advised to capture new gene–disease associations [62,63].

When “red-flag” features are present—such as developmental regression, episodic metabolic crises, multisystem involvement, or parental consanguinity—metabolic testing should be incorporated, following a “screen-for-treatables” model. Neuroimaging (MRI) is recommended in the presence of neurological abnormalities, seizures, or atypical head growth, while EEG should be reserved for suspected epilepsy or unexplained paroxysmal episodes [64,65].

Environmental and infectious factors must also be addressed. In infants with suspicious clinical features, congenital cytomegalovirus (cCMV) should be confirmed or excluded promptly because of its implications for antiviral therapy and auditory follow-up. In at-risk populations, blood lead screening should follow the CDC’s 3.5 µg/dL reference threshold to guide early intervention.

Above all, developmental therapy and family support should begin as soon as delays are identified—well before the etiologic investigation is complete. This parallel, integrated approach—combining comprehensive assessment, tiered investigations, and early therapeutic engagement—maximizes developmental potential while minimizing diagnostic delays [66,67].

**Figure 5:**
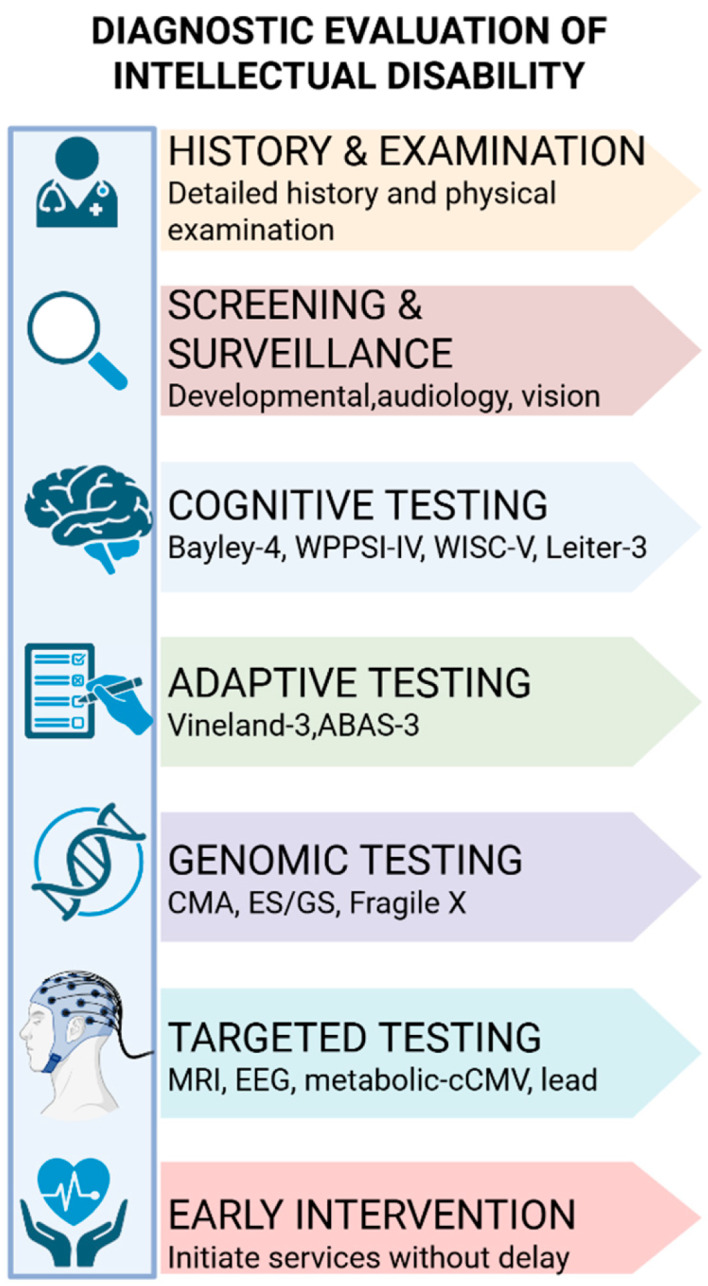
Schemes follow the same formatting. A structured diagnostic pathway is essential to ensure accuracy, efficiency, and timelines in evaluating intellectual disability. The process begins with a comprehensive history and physical examination. Created in BioRender. Babiker, R. (2025) https://BioRender.com/9ygdqw0.

Figure 5 outlines the structured, stepwise approach from clinical history to targeted investigations, clarifying the parallel processes of evaluation and early intervention that optimize developmental outcomes.

## 3. Methodological and Ethical Considerations in Assessment

**Validity and Fairness:**

Standardized measures of intellectual and adaptive functioning are indispensable for diagnosis but not without limitations. All IQ and adaptive behavior instruments contain inherent measurement error, and results may be influenced by rater bias, cultural or linguistic mismatch, or limited familiarity with formal testing contexts [68].

Standardized measures of intellectual and adaptive functioning are indispensable for diagnosis but not without limitations. All IQ and adaptive behavior instruments contain inherent measurement error, and results may be influenced by rater bias, cultural or linguistic mismatch, or limited familiarity with formal testing contexts. It is also important to recognize that most standardized IQ tests are culturally bound and normed primarily on Western populations. Consequently, they may have limited validity or availability in many regions, underscoring the urgent need for culturally adapted measures and context-sensitive interpretation to ensure diagnostic fairness worldwide [68,69].

Ongoing psychometric discussions—such as those surrounding the structural properties of the Vineland-3—underscore the need for cautious and contextual interpretation. Clinicians are therefore advised to synthesize findings from multiple informants and environments rather than relying on rigid numerical cut-offs. The DSM-5-TR’s deliberate shift toward adaptive functioning was designed, in part, to minimize the risk of misclassification arising from IQ-based thresholds that fail to capture real-world competence [70].

**Equity:**

Access to comprehensive diagnostic evaluation is uneven across populations. Socioeconomic disparities, geographic isolation, and fragmented healthcare infrastructure frequently delay both assessment and intervention, disproportionately affecting children in underserved settings. Embedding developmental surveillance and screening within primary care—an approach strongly recommended by the American Academy of Pediatrics—and facilitating first-line genetic testing at the point of initial clinical suspicion can significantly reduce these inequities. Culturally equitable assessment also requires sensitivity to linguistic and contextual variation, ensuring that diagnostic tools, normative data, and interpretive frameworks reflect the child’s sociocultural background [71].

**Communication:**

Ethical diagnostic practice extends beyond accuracy to the quality of communication. Families should receive results in clear, empathetic, and family-centered language that highlights both the child’s strengths and areas of need, accompanied by practical next steps. Guidance should encompass educational rights, access to community-based services, and long-term planning, with recommendations revisited periodically as the child develops. Transparent, compassionate communication reduces stigma, promotes family coping, and fosters collaborative decision-making [72].

### Emerging Frontiers Likely to Raise Diagnostic Yield and Utility

Rapid technological and clinical advances are reshaping the diagnostic landscape for intellectual disabilities. Genome sequencing and reanalysis pipelines represent a leading frontier: as variant databases expand and bioinformatic tools mature, periodic reanalysis of previously nondiagnostic exomes or genomes is increasingly yielding new insights. Whole-genome sequencing (GS) extends beyond exome sequencing (ES) by detecting structural and noncoding variants, thereby closing long-standing diagnostic gaps [73].

Condition-specific newborn screening and targeted early detection offer another promising direction. Several jurisdictions are now piloting universal or risk-based newborn screening for congenital cytomegalovirus (CMV)—the most common congenital infection worldwide. Early identification enables timely antiviral therapy and structured audiologic monitoring, with broader adoption anticipated as cost-effectiveness evidence grows. Comparable strategies may eventually extend to other genetic and metabolic disorders as novel therapeutic interventions become available [74].

Digital phenotyping and remote assessment are also expanding diagnostic accessibility. Tele-practice adaptations of adaptive behavior scales, such as the Vineland-3, demonstrated strong feasibility and scalability during the COVID-19 pandemic. Hybrid diagnostic models—combining in-clinic standardized testing with caregiver-reported data and digital behavioral metrics—are increasingly validated, reducing access barriers while maintaining psychometric integrity [75].

Gene-directed therapies further highlight the transformative impact of precise molecular diagnosis. In monogenic neurodevelopmental syndromes, genetic findings already guide condition-specific monitoring and targeted interventions—for example, mTOR inhibition in tuberous sclerosis. The expanding range of targeted therapies underscores the urgency of early and accurate diagnosis, as molecular confirmation determines eligibility for disease-modifying treatments and participation in clinical trials [76,77].

**Figure 6:**
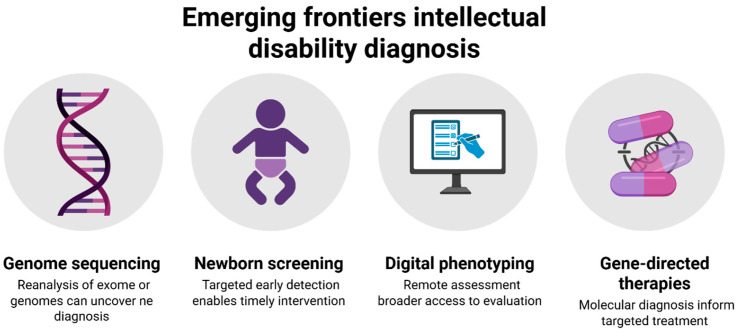
This is a figure that illustrates a future in which diagnosis is not static but dynamic—refined through genomic science, digital innovation, and therapeutic translation—ultimately linking precision in classification with opportunities for intervention. Created in BioRender. Babiker, R. (2025) https://BioRender.com/u7e3m4m.

Together, these emerging frontiers depict a future in which diagnosis is dynamic rather than static—continually refined through genomic discovery, digital innovation, and translational therapeutics. Precision in classification will increasingly intersect with precision in intervention, ensuring that diagnostic advances translate directly into improved outcomes. (Figure 6 conceptually integrates these frontiers, linking genetic reanalysis, digital phenotyping, and gene-targeted therapies within a unified precision-diagnosis framework.)

Figure 6 conceptually links ongoing advances—genomic reanalysis, digital phenotyping, and gene-directed therapies—to a dynamic, precision-based model of diagnosis that continually evolves with scientific progress.

## 4. Future Directions

### 4.1. Personalized In Vivo Gene Editing

A recent landmark—the first personalized CRISPR-based therapy delivered via lipid nanoparticles to treat a newborn with severe CPS1 deficiency—demonstrates how precision gene editing can be rapidly tailored to individual genetic conditions. This modular, patient-specific approach may soon translate into early, etiologically targeted treatments for select monogenic intellectual disability syndromes [78].

### 4.2. Affordable Gene Therapy Scale-Up

The Innovative Genomics Institute (IGI) is actively developing strategies for reducing the cost of genetic and genome-editing therapies by up to tenfold through novel funding models, manufacturing innovations, and alternative licensing strategies. This cost paradigm shift could make cutting-edge genomic treatments far more accessible across diverse healthcare settings [79].

### 4.3. Gene-Editing for Chromosome-Level Disorders

CRISPR-Cas9 has been recently applied to remove the extra copy of chromosome 21 in cells from individuals with Down syndrome, normalizing gene expression and cellular growth patterns. While ethically and technically complex, such approaches suggest a future in which previously intractable aneuploid conditions might one day be addressed at a cellular or even organismal level [80].

### 4.4. New FDA-Approved Oral Therapy for Phenylketonuria (PKU)

In July 2025, the U.S. FDA approved Sephience, a novel oral drug that significantly increases phenylalanine hydroxylase (PAH) activity, lowering blood phenylalanine by ~63% in PKU patients and easing dietary restrictions. PKU is a classic cause of intellectual disability when untreated, so this advancement underscores how a diagnostic label can now directly guide a small-molecule intervention with major quality-of-life implications.

### 4.5. Integrating Emerging Therapeutics with Diagnostic Frameworks

Collectively, these emerging innovations—from gene editing and affordable genomic therapies to digital phenotyping and condition-specific screening—represent the translational continuum of the diagnostic principles outlined earlier in this review. The precision emphasized in DSM-5-TR, ICD-11, and the Clinical Descriptions and Diagnostic Requirements (CDDR)—with their focus on adaptive functioning, developmental context, and equitable access—forms the foundation upon which these novel interventions rest. Accurate and early diagnosis enables timely enrollment in gene-based trials, identification of targetable pathways, and informed family counseling, transforming the diagnostic process from a static classification into a gateway for precision therapy. Thus, advances in diagnostic accuracy and therapeutic innovation are mutually reinforcing, converging toward a unified vision of personalized, equitable care for children with intellectual disabilities.

## 5. Conclusions

Childhood intellectual disability is both common and heterogeneous, yet importantly, it is also actionable. Contemporary nosologies such as the DSM-5-TR and ICD-11 have shifted diagnostic emphasis from IQ thresholds to adaptive functioning, aligning classification more closely with lived abilities, support needs, and real-world outcomes. This conceptual change underscores the centrality of functional skills in guiding eligibility for services and intervention planning.

The diagnostic pathway that delivers the greatest clinical gains is also the most parsimonious: begin with vigilant listening, developmental surveillance, and early screening; evaluate cognition and adaptive behavior with age-appropriate, culturally fair tools; and implement genomic testing—preferably exome or genome sequencing—early in the process, supplemented by phenotype-driven targeted studies. Additional investigations, including metabolic screening, neuroimaging, EEG, and environmental or teratogenic evaluations, should be undertaken selectively when red flags are present. Crucially, early intervention must commence as soon as developmental delays are detected, without waiting for the full etiologic work-up to conclude.

When applied consistently, this framework shortens the diagnostic odyssey, reduces inequities in access to care, and transforms diagnosis from a static label into a dynamic platform for personalized surveillance, timely intervention, and family-centered planning. Looking ahead, the integration of genomic medicine, digital phenotyping, and emerging gene-directed therapies holds the potential not only to refine diagnostic yield but also to open pathways for targeted treatments. By advancing diagnostic accuracy and ensuring equitable access, clinicians and policymakers can measurably improve the developmental trajectories and quality of life of children and families affected by intellectual disability.

## Data Availability

Not applicable.

## Abbreviations

The following abbreviations are used in this manuscript:

ID	Intellectual disability
DSM-5-TR	Diagnostic and Statistical Manual of Mental Disorders, Fifth Edition, Text Revision
ICD-11	International Classification of Diseases, 11th Revision
CDDR	Clinical Descriptions and Diagnostic Requirements
GBD	Global Burden Disease
AAP	American Academy of Pediatrics
ASD	Autism Spectrum Disorder
FASD	Fatal alcohol spectrum disorders
WPPSI	Wechsler Preschool and Primary Scale of Intelligence—Fourth Edition
WISC-V	Wechsler Intelligence Scale for Children—Fifth Edition
Vineland-3	Vineland Adaptive Behavior Scales, Third Edition
ABAS-3	Adaptive Behavior Assessment System, Third Edition
ACMG	American College of Medical Genetics and Genomics
CMA	Chromosomal Microarray
EEG	Electroencephalography
CMV	Congenital cytomegalovirus
ADHD	Attention-deficit/hyperactivity disorder
IGI	Innovative Genomics Institute
